# VEGFR2-specific FnCAR effectively redirects the cytotoxic activity of T cells and YT NK cells

**DOI:** 10.18632/oncotarget.24078

**Published:** 2018-01-09

**Authors:** Sergey V. Kulemzin, Andrey A. Gorchakov, Anton N. Chikaev, Valeriya V. Kuznetsova, Olga Y. Volkova, Daria A. Matvienko, Alexey V. Petukhov, Andrey Y. Zaritskey, Alexandr V. Taranin

**Affiliations:** ^1^ Institute of Molecular and Cellular Biology SB RAS, Novosibirsk, Russia; ^2^ Novosibirsk State University, Novosibirsk, Russia; ^3^ Institute of Hematology, Almazov National Medical Research Centre, St Petersburg, Russia

**Keywords:** chimeric antigen receptors, NK cells, Fn3, VEGFR2, FnCAR

## Abstract

T and NK cells armed with chimeric antigen receptors (CAR) are promising tools for the specific elimination of cancer cells. In most CAR designs implemented to date, the recognition of target cells is mediated by single-chain variable fragments (scFvs) derived from murine monoclonal antibodies. This format, however, has a number of limitations, including its relatively large size and potential immunogenicity in humans. In this study, we explored the feasibility of using human fibronectin type III domains (Fn3) as the antigen recognition domain in CARs. Human Fn3 domains have lower predicted immunogenicity compared to mouse-derived sequences, and a reduced molecular weight compared to scFvs. We created a functional CAR using a VEGFR2-specific Fn3 module replacing the conventional scFv. The resulting FnCAR specifically potentiates the cytotoxic activity of human T cells and YT NK cells in the presence of VEGFR2-positive targets. These findings demonstrate that Fn3 domains can be used in CARs for antigen recognition.

## INTRODUCTION

T-cells expressing CD19-specific Chimeric Antigen Receptors (CARs) demonstrated remarkable anti-tumor activity in patients with advanced B-cell malignancies [1–6], and other CARs are currently being evaluated in the clinical and preclinical settings. The antigen-recognition domains of most CARs contain scFv (single-chain fragment variable) sequences derived from well-characterized monoclonal antibodies specific to tumor-associated surface antigens. While the scFv-derived targeting domains inherit the specificity and versatility of monoclonal antibodies, they also have a number of important limitations. The availability of fully human scFv sequences is still scarce, and reducing the immunogenicity of murine scFvs by grafting CDRs onto human framework sequences often results in unpredictable changes in the folding or antigen recognition capacity of the resulting scFvs. Furthermore, the correct folding of an scFv requires the proper formation of intramolecular disulfide bonds that may complicate the design of multispecific CARs incorporating two or more scFvs. Finally, the framework regions of scFvs have been shown to frequently produce CARs that are prone to tonic signaling—an unwanted effect that results in the premature exhaustion of CAR T-cells [7].

The 10th type III domain of human fibronectin 1 (Fn3) is a small globular module comprising seven antiparallel beta-strands folded as two beta-sheets [8]. Surface-exposed loops connecting these beta-strands generally have little influence on the structural stability of the Fn3s and, therefore, their randomization can be used to obtain Fn3 proteins of the desired binding specificity and affinity. Thus, highly complex Fn3 libraries have been created and successfully screened using phage, yeast, and ribosomal displays to select the specific variants capable of binding target proteins with high affinity at the picomolar to low nanomolar range [9–11]. Of these, CD20-, CEA-, EGFR-, IGF-1R, and VEGFR2-specific Fn3s have been validated in pre-clinical and clinical settings as promising imaging and/or therapeutic molecules [12–15]. While Fn3s are structurally homologous to the variable fragments of antibodies, they are more compact (~94 aa), devoid of intramolecular disulfide bonds and are naturally present as multidomain chains. In addition, several engineered Fn3s were found to recognize concave epitopes that are typically missed by scFvs [10, 16]. These properties make Fn3-based domains an attractive option to the standard scFv-derived modules, potentially allowing for the broadening of the repertoire of targetable epitopes and streamlining the design of multi-specific CARs.

In this proof-of-concept study, we designed and characterized a VEGFR2-specific CAR with the antigen-recognition moiety derived from the Fn3-scaffold. We show that FnCARs are functional, as they drive the specific activation of human T cells. In addition, the expression of these FnCARs promotes the specific cytotoxicity of primary human T cells and YT NK cells *in vitro*. These results support the feasibility of using Fn3 domains in CARs.

## RESULTS

### Construction of VEGFR2-specific FnCARs

**Figure 1:**
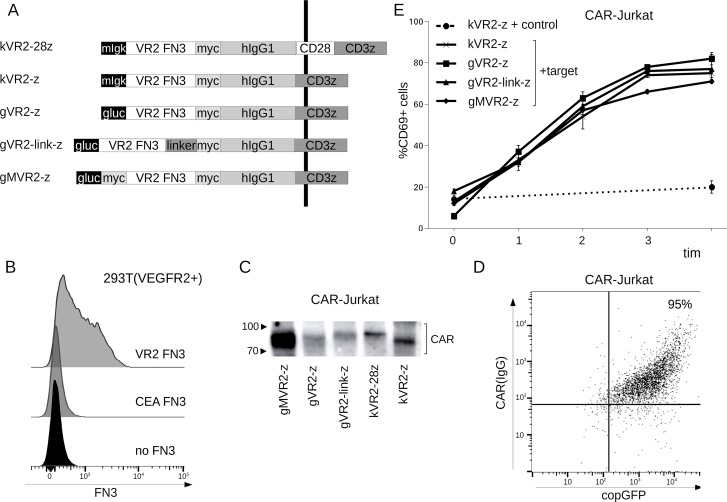
(**A**) Schematic of CAR constructs containing VEGFR2-specific Fn3-based antigen-recognition module. CARs obtained encompass leader sequences from either mIgk or Gaussia princeps luciferase (Gluc), VEGFR2-specific Fn3 sequence (VR2 FN3), myc epitope tag, hIgG1 spacer region (hinge-CH2-CH3 domains), CD28 region (transmembrane and signaling sequences), and CD3ζ region (transmembrane and/or signaling sequences). The vertical black line denotes the cell membrane. (**B**) FACS detection of VEGFR2 expression on the surface of HEK293T(VEGFR2+) cells stained with either recombinant FLAG-tagged Fn(VEGFR2)—VR2 FN3, FLAG-tagged Fn3 of irrelevant specificity—CEA FN3 [15], or left unstained. (**C**) Western blot detection of FnCAR expression in transduced Jurkat cells (anti-myc). (**D**) flow cytometry surface staining of kVR2-28z FnCAR-expressing Jurkat cells (becoming copGFP+ upon transduction) with anti-hinge (IgG-specific APC-labeled) conjugates. (**E**) Expression of the activation marker CD69 on CAR-Jurkat cells incubated with HEK293T(VEGFR2+) target cells or isogenic control cells (HEK293T) for the times indicated.

We used a modular pCDH-based lentiviral system [17] in order to generate a series of FnCARs specific for the human VEGFR2. Antigen-recognition module of the FnCARs obtained was based on the clone K10 described previously [13] whereas IRES-linked copGFP reporter allowed convenient detection of the transduced cells. Testing several combinations of CAR modules was warranted to ensure that the FnCARs are efficiently transported to the cell surface and are easily detectable. Specifically, several FnCAR variants were constructed that differed in the signal peptides (*Gaussia princeps* luciferase vs. mouse Igk), position of the myc-epitope (central and/or N-terminal), and cytoplasmic signaling sequences (CD3z *vs.* CD28+CD3z). All FnCARs shared the same spacer region derived from the human IgG1 (hinge-CH2-CH3) (Figure 1A).

### FnCARs are expressed on the surface of Jurkat cells

First, we verified the specificity of the VEGFR2-specific Fn3 used. This Fn3 was produced in recombinant form in *E. coli* as a fusion with 2xStrep-2xFLAG-6xHis tag and used for staining 293T cells designed to stably express VEGFR2 (Supplementary Figure 1). A specific anti-FLAG signal was observed only for VEGFR2-expressing cells, but not in the isogenic negative controls (Figure 1B), which cross-validates both the Fn3(VEGFR2) and the target cells. Next, we asked whether FnCARs could be produced in a Jurkat T-cell line and, if so, whether they become surface expressed. The constructs obtained were used for producing VSV-G pseudotyped lentiviral particles that were transduced into Jurkat cells. Our Western blot and FACS data confirm that FnCARs are successfully synthesized by the transduced Jurkat cells at comparable levels (Figure 1C) and that they are indeed expressed on the cell surface, as assayed by anti-IgG1 staining (Figure 1D, shown for kVR2-28z).

### FnCARs can activate Jurkat T cells

Having established the specificity and surface expression of FnCARs, we proceeded to test their functionality. FnCAR-Jurkat cells display specific and rapid activation (Figure 1E) when incubated with the appropriate target cells (VEGFR2+, solid lines) but not with isogenic control cells (VEGFR2-, dashed line) as assayed by the upregulation of an early activation marker CD69. Our data thus indicate that regardless of the position of the myc epitope or the signal peptide used, FnCARs show robust activation properties in the context of Jurkat cells.

### FnCARs are functional in the context of primary human T cells

Although Jurkat cells are routinely used for rapid and convenient testing of different CAR designs, they are not cytotoxic. Hence, we asked whether FnCARs would be expressed by the transduced primary human T cells and, if so, whether this would result in their VEGFR2-specific activation and cytotoxicity.

**Figure 2:**
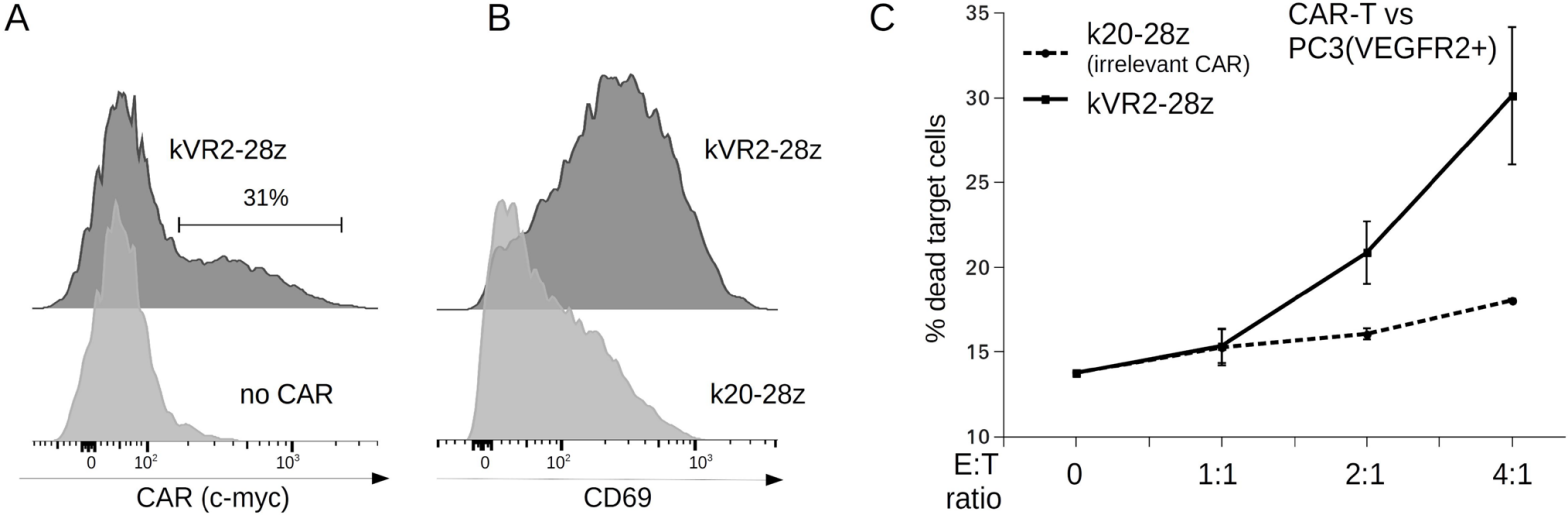
(**A**) Flow cytometry detection of CAR expression on the surface of transduced FnCAR T cells, as assayed by anti-myc staining. (**B**) VEGFR2-specific FnCAR-T cells but not irrelevant CAR-T cells (gated by the expression of CAR) become activated (CD69+) upon incubation with target PC3(VEGFR2+) cells. (**C**) PC3(VEGFR2+) target cell killing by VEGFR2-specific FnCAR-T cells (note that only ~30% of effector T cell population is FnCAR-positive, (A)), but not by irrelevant CD20-specific k20-28z CAR-T cells.

Given that all of the FnCAR designs tested hereinabove behaved very similarly, we picked a single representative second-generation FnCAR variant, kVR2-28z. Much as was observed for the FnCAR-Jurkat cells, transduced primary human T cells readily expressed kVR2-28z (Figure 2A) and became specifically activated upon co-incubation with VEGFR2+ cell targets, as manifested by the upregulated CD69+ expression (Figure 2B). Accordingly, FnCAR-T cells were moderately cytotoxic toward VEGFR+ cell targets (Figure 2C).

### FnCARs are functional when expressed by a human NK-cell line, YT

Human NK cell lines (NK-92, YTS, KHYG-1, etc.) represent an attractive platform for creating allogeneic CAR-NK cell lines that can be universally administered to cancer patients in an “off-the-shelf” format without the need for patient-specific manufacture [18]. Therefore, we turned to one such NK-cell line, YT [19], which offers the advantage of easy transduction and IL2-independence, for exploring whether our FnCARs can endow them with VEGFR2-specific cytotoxicity.

**Figure 3:**
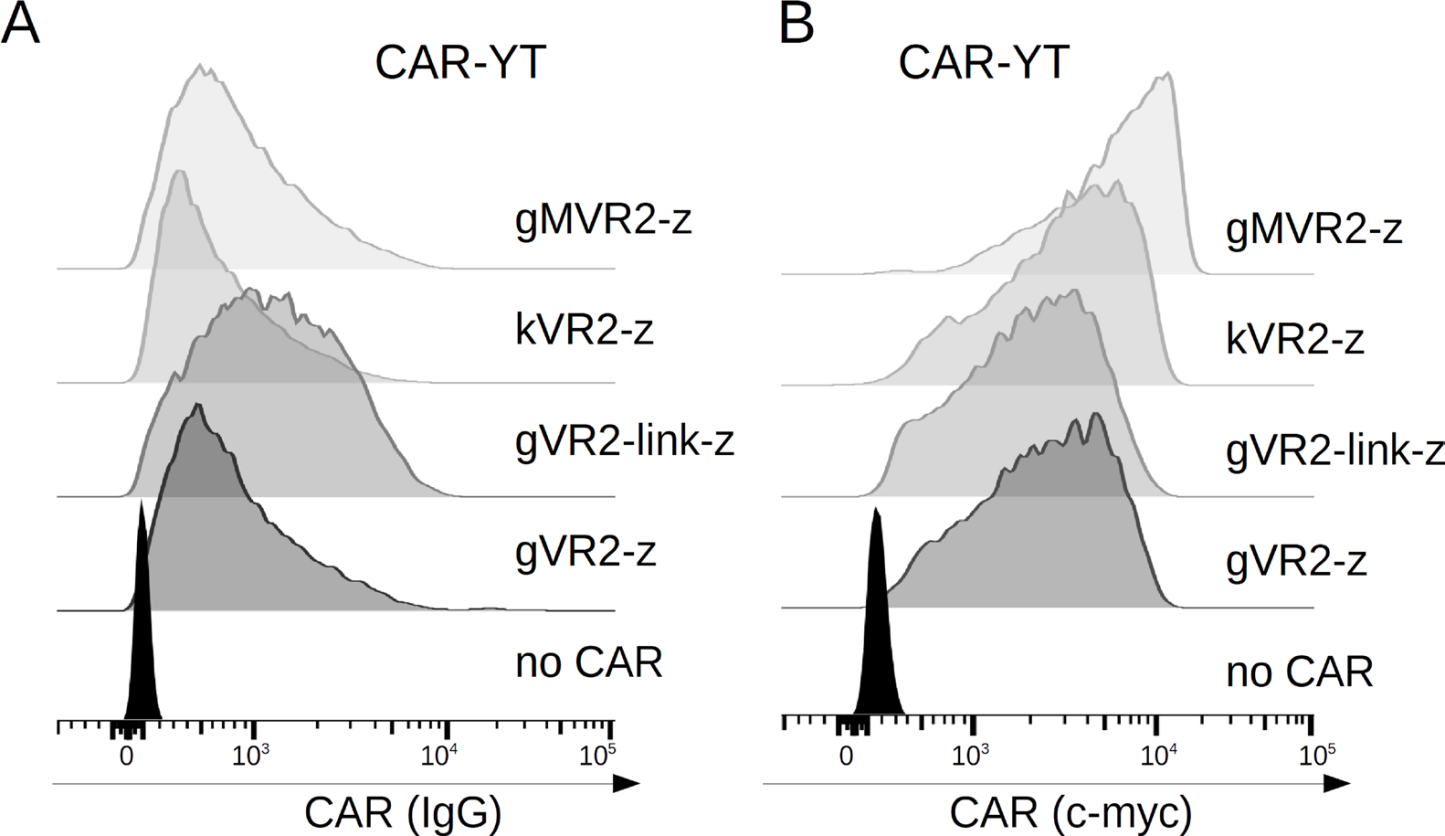
FACS histograms showing CAR expression on the surface of YT cells, as assayed by anti-IgG (**A**) or anti-myc (**B**) staining. MFI values (top-down) 1146, 858, 1735, 1278, 148 (A), and 8286, 6465, 5091, 5218,789 (B).

First, we ascertained the surface expression of FnCARs by YT cells. Similarly to FnCAR-Jurkat cells, FnCAR expression was readily detectable on transduced YT cells (Figure 3A). Notably, the incorporation of a flexible linker between Fn3 and IgG1 domains resulted in a modest increase in the surface expression of FnCARs (Figure 3A). In addition, a side-by-side comparison of transduced FnCAR-YT cells stained with either myc- or IgG-specific conjugates shows that placing the myc epitope between the Fn3 and IgG1 modules was somewhat suboptimal for flow cytometry purposes: although detectable, it appeared partially shielded from the interaction with anti-myc antibodies. Instead, when the myc epitope was positioned between the signal peptide and the Fn3 module, this enabled the robust detection of the CAR (Figure 3B).

**Figure 4:**
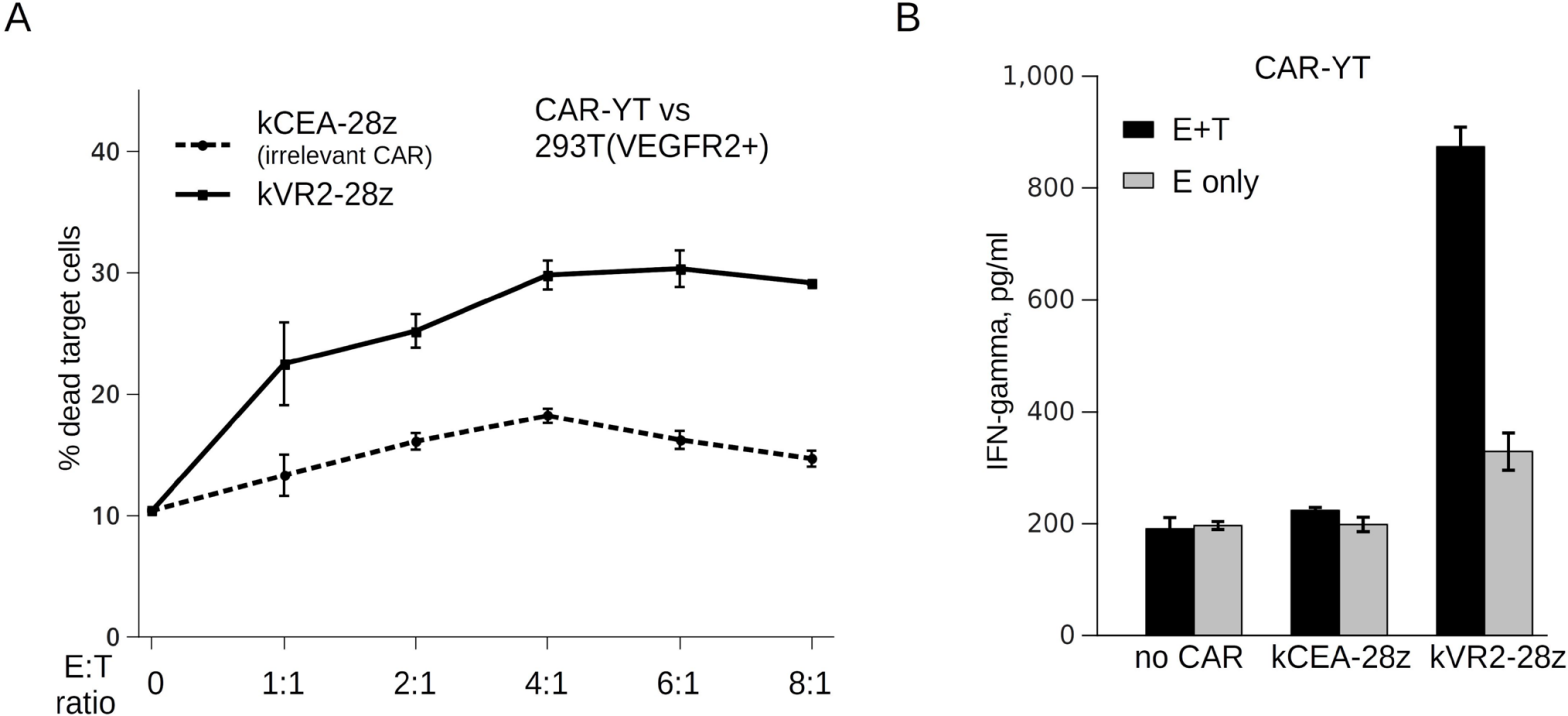
(**A**) HEK293T(VEGFR2+) target cell killing by VEGFR2-specific FnCAR-YT cells or YT cells transduced with an irrelevant Fn3(CEA)CAR, at the indicated E:T ratios. (**B**) IFNgamma release by FnCAR-transduced or non-transduced YT cells in the presence (black bars) or absence (grey bars) of target HEK(VEGFR2+) cells. kCEA-28z CAR targeting an irrelevant surface molecule, CEA, was used as a negative control.

Next, we asked whether FnCAR-YT cells were cytotoxic. kVR2-28z CAR was selected to measure the FnCAR-mediated cytotoxicity of YT cells. Indeed, FnCAR-YTs killed VEGFR2-expressing HEK293T cells and spared VEGFR2-negative cells even at low E:T ratios (Figure 4A).

In order to determine whether the observed enhanced cytotoxicity of FnCAR YT cells was also accompanied with increased and specific cytokine release, we analyzed the IFNgamma secretion by FnCAR YT cells co-cultured with target (VEGFR2+) or non-target (VEGFR2-) cells. As a control, irrelevant FnCAR YT cells recognizing a distinct molecule (CEA) and CARless unmodified YT cells were included in the experiment. Much as was expected, FnCAR YT cells displayed a pronounced and specific IFNgamma release upon FnCAR engagement by cognate VEGFR2-epxressing cells (Figure 4B). Taken together, these data indicate that engineering FnCARs into NK cells may significantly enhance their effector functions in response to the appropriate target cells, which warrants further testing of this platform in pre-clinical models.

## DISCUSSION

Current progress in CAR T-cell therapies is hampered by the lack of truly cancer-specific surface markers targetable with scFv-based CARs. This technological bottleneck is partially addressed by designing different variants of “smart” CARs that are capable of discriminating combinations of antigens from single-antigen carrying cells [20–25]. In fact, CAR platform is plagued by the same issue that has been long known for monoclonal antibody-based therapies, i.e. tumor escape as a result of immunoediting. Hence, several groups have focused their efforts on the design of bi-specific CARs [26–28]. Both issues converge in the necessity of developing alternative formats of compact antigen-recognition modules that would help expand the repertoire of targetable cancer cell surface markers and would also be amenable for structure-guided multimerization. Several non-scFv formats that partially fit this description have been tested in the CAR context, which include VHHs (nanobodies) [29–32], VLRs [33], DARPins [34], and affinity peptides [35–37]. At present, neither of them has been reported to be successfully used for creating bi- or multispecific CARs. Importantly, these alternative antigen-recognition molecules are of non-human origin, which may limit their successful translation into the clinic due to immunogenicity concerns. The vast majority of the scFv-based CARs tested in clinical trials are murine or humanized. The recent development of fully human scFv-based CARs against several hematological [38–40] and solid [41, 42] cancer cell targets has proven effective in animal models and warrants the exploration of alternative scaffolds based on the human sequences as antigen-recognition modules in CARs. Fn3s represent one such option. These proteins are derived from the 10^th^ type III domain of the human protein fibronectin 1. Fn3s share significant structural homology with variable fragments of antibodies in that they are formed by a sandwich of two antiparallel beta-sheets connected with solvent-accessible CDR-like loops [8, 43]. These loops can be readily diversified and used for the identification of affinity binders to the targets of interest [44]. In contrast to antibody variable fragments, however, Fn3s generally lack intramolecular disulfide bonds or free cysteines. Furthermore, much like VHHs, VLRs, and DARPins, Fn3s are more compact than scFvs and are amenable to tandemization [14]. We envisage that this may help create bi-specific CARs having antigen-recognition modules comprising Fn3s or combinations of Fn3s with scFvs and other scaffolds. Finally, a number of Fn3s developed to date have been shown to bind concave epitopes that are typically inaccessible to bulkier paratopes of scFvs [16]. In our opinion, this feature of Fn3s may translate into a greater choice of epitopes targetable with FnCARs.

In our proof-of-concept study, we asked whether Fn3s can be used as the antigen-recognition modules of CARs and demonstrate that this is indeed the case, consistent with the multitude of non-scFv CARs developed to date. A panel of VEGFR2-specific FnCARs having minor structural differences was successfully designed and expressed in Jurkat, primary T, and YT cells. FnCARs endowed the cells with the ability to become specifically activated when confronted with VEGFR2-positive target cells. In the context of primary human T cells and the YT NK-cell line, FnCAR expression rendered them cytolytic against VEGFR2-expressing targets but not VEGFR2-negative controls, which was accompanied with a significant increase in IFNgamma release. Thus, we demonstrate that FnCARs are fully functional in primary T cells and YT cells. Finally, recent studies have implicated the framework regions of scFvs as responsible for the effect of ligand-independent tonic signaling in CAR-T cells, which may result in their slow expansion *ex vivo*, premature exhaustion, and suboptimal persistence once transferred into the patients [7]. We hypothesize that non-scFv formats, such as VHHs, VLRs, DARPins, and Fn3s may help address this problem and afford improved CAR designs.

## MATERIALS AND METHODS

### Cell lines and primary T cells

Jurkat (clone E6-1) cells were purchased from American Type Culture Collection (ATCC), YT cells were kindly provided by Dr. Aleksandr V. Filatov. HEK293T(VEGFR2+) cells were obtained by infecting HEK293T cells with VEGFR2-encoding lentivirus (pCDH-VEGFR2deltaC-IRES-copGFP) followed by single cell cloning. PC3(VEGFR2+) cells were obtained in a similar way. All of the cell lines were maintained in IMDM supplemented with 10% FCS (HyClone), 100 U/ml penicillin, and 100 ug/ml streptomycin (Sigma).

Primary human PBMCs were isolated from a healthy donor after informed consent in accordance with the Declaration of Helsinki. T cells were purified from PBMCs using Dynabeads Untouched Human T Cells Kit (ThermoFisher) and activated for 24 h using Human T-activator CD3/CD28 Dynabeads (ThermoFisher). The cells were then kept in a medium supplemented with 50 U/ml human IL2 (Peprotech).

### Cloning of FnCAR constructs

Lentiviral vector pCDH was used as a backbone for the construct design. Based on this vector, first- and second-generation CAR constructs encoding mIgk or Gaussia luciferase signal sequence, myc epitope, hIgG1(hinge-CH2-CH3), CD3z or CD3z+CD28 sequences were obtained (GenBank accession numbers KX757243, KX757244, KX757252, KX757253) [17]. Sequence coding for the Fn3(VEGFR2) module (K10 clone) was extracted from the literature data [13], produced by gene synthesis (Biomatik, Canada) and cloned between unique AgeI and BamHI sites of the above vectors in frame with the rest of the CAR modules. Thus, kVR2-28z, kVR2-z, and gVR2-z constructs were obtained. Next, we explored whether the incorporation of a flexible (GS)_11_ linker would create a more active CAR and whether adding a myc-epitope to the N-terminus of Fn3 module would be well-tolerated (as recombinant Fn3s have been reported to be sensitive to the changes at their N-termini [13]). A linker sequence was introduced between the Fn3(VEGFR2)- and myc-coding sequences using PCR to form gVR-link-z construct. A duplex of oligos encoding N-myc tag was ligated into the AgeI site of gVR2-z between the Gaussia leader and the Fn3 to obtain gMVR2-z.

### Lentivirus production and transduction

HEK293T cells were co-transfected with FnCAR- or CAR-encoding lentiviral constructs and packaging plasmids pMD2.G and psPAX2 using the calcium phosphate transfection procedure. Two days later, supernatants were filtered using 0.45 um PES filters. Titers of all viral supernatants were at least 10^7 infective particles per ml. Jurkat, primary human T cells or YT cells were transduced with viral supernatants by spinoculation method [45] in the presence of 2 ug/ml polybrene (Jurkat and YT cells) or 10 ug/ml protamine sulfate (primary T cells). Jurkat and YT cells were consistently infected at efficiencies above 70%, whereas primary human T cells were typically transduced at 30–40% (Figure 2A). Cells infected with different viral samples displayed similar MFI values for copGFP marker, which indicated that a comparable MOI was achieved.

### Activation assay

One hundred thousand FnCAR Jurkat cells were incubated with an equal number of HEK293T(VEGFR2+) or with wild-type HEK293T(VEGFR2-) cells in 24-well plates (TPP # 92124) for 4 h in a CO2 incubator. Alternatively, 0.1 million FnCAR-T cells were incubated with an equal number of PC3(VEGFR2+) cells as the targets for 4 h (irrelevant CD20-specific k20-28z CAR T cells were used as a negative control). Immediately after incubation, the cells were stained with anti-CD69 antibodies (PE-labeled clone FN50, eBioscience) and analyzed with BD FACSCanto II cytometer.

### Fn3 specificity validation and surface CAR expression analysis

Soluble VEGFR2-specific 2xStrep-2xFLAG-6xHis-tag labeled Fn3 was produced in *E. coli* and purified using Ni-NTA agarose (Qiagen) according to the manufacturer’s protocol. Briefly, 1 g of *E. coli* pellet was resuspended in 10 ml of lysis buffer (20 mM Tris-HCl pH 8.0, 350 mM NaCl, 20 mM Imidazole, 5 mM beta-ME) and disrupted by French press G-M (Glen Mills). Lysate was clarified by centrifugation and applied to 1 ml of Ni-NTA agarose. The resin was then washed with 5 volumes of lysis buffer and protein was eluted with elution buffer (20 mM Tris-HCl pH 8.0, 350 mM NaCl, 250 mM Imidazole, 5 mM beta-ME). Eluate was dialyzed against PBS and concentrated to 1 mg/ml. HEK293T(VEGFR2+) cells were incubated for 30 min with 1 ug/ml of soluble Fn3(VEGFR2) in PBS with 1% FCS, washed twice with PBS, and stained with anti-FLAG antibodies (clone FG4R, eBioscience). To measure surface CAR expression, either anti-c-myc antibodies (clone 9E10, eBioscience) or anti-IgG-APC conjugates (709-606-098, Jackson ImmunoResearch) were used. Detection of myc- or FLAG-stained cells was performed using PE-anti-mouse IgG conjugates. All FACS gates were set using untransduced cells or cells with an omitted primary antibody.

### Cytotoxicity assay and IFNgamma release assay

For the detection of FnCAR-mediated cell lysis, FACS-based protocol was employed [46]. Briefly, target HEK293T(VEGFR2+) or PC3(VEGFR2+) cells were labelled with Cell Proliferation Dye eFluor® 670 (eBioscience), washed, and incubated with Fn3(VEGFR2)CAR-expressing effector cells or control irrelevant CAR-expressing cells (T or YT) at various E:T ratios for 4 hours. Then, the cells were stained with 7AAD (1 ug/ml) and analyzed by flow cytometry. The percentage of dead target cells was calculated as follows: 100% * (7AAD+, eFluor+)_count_/(total eFluor+)_count_.

In order to measure IFNgamma release, Fn3(VEGFR2)CAR YT, irrelevant Fn3(CEA)CAR YT or wild-type YT cells were incubated with target HEK293T(VEGFR2+) or control HEK293T(VEGFR2-) cells at a 1:1 ratio for 4 hours. Immediately after incubation, cell culture supernatants were filtered through 0.45 um filters and IFNgamma concentration in the supernatants was measured in duplicates using ELISA (IFA-best kit, Vektor-best, Russia).

## SUPPLEMENTARY MATERIALS AND FIGURE



## Abbreviations

CAR: chimeric antigen receptor
CDR: complementarity-determining region
CEA: carcinoembryonic antigen (CEACAM5)
copGFP: green fluorescent protein 2 from the copepod *Pontellina plumata*
DARPins: designed ankyrin repeat proteins
Fn3: fibronectin type III domain
FnCAR: Fn3-based chimeric antigen receptor
IRES: internal ribosome entry site
MFI: mean fluorescence intensity
MOI: multiplicity of infection
NK: natural killer
scFv: single-chain fragment variable
VEGFR2: vascular endothelial growth factor receptor 2 (KDR: CD309)
VHHs: camelid single-domain antibodies
VLRs: sea lamprey variable lymphocyte receptors.
