# Supplementary material for: VEGFR2-specific FnCAR effectively redirects the cytotoxic activity of T cells and YT NK cells
Source: Oncotarget. 2018 Jan 9;9(10):9021–9. doi: 10.18632/oncotarget.24078 (PMC5823625; doi:10.18632/oncotarget.24078)
Supplement: Supplementary file 1 [file oncotarget-09-9021-s001.pdf]

VEGFR2-specific FnCAR effectively redirects the cytotoxic activity of T cells and YT NK cells

SUPPLEMENTARY MATERIALS

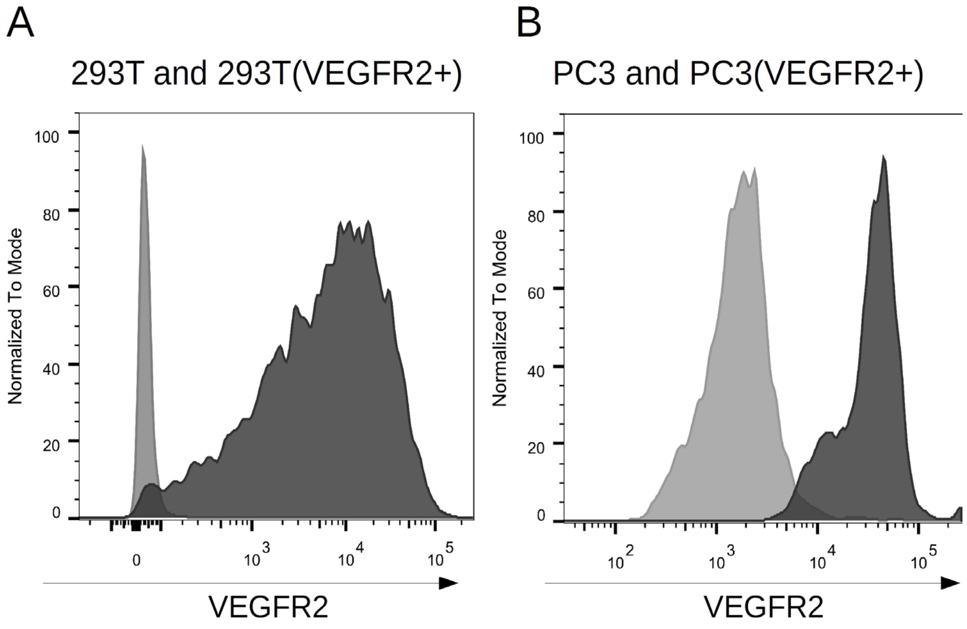

Supplementary Figure 1: HEK293T (**A**) and PC3 (**B**) cells were stably transduced to express VEGFR2 lacking the intracellular domain and stained with anti-VEGFR2 mAb from Miltenyi (130-093-603). The robust surface expression of VEGFR2 is detectable in transduced but not in parental non-transduced cells.
